# TRIP13 promotes the cell proliferation, migration and invasion of glioblastoma through the FBXW7/c-MYC axis

**DOI:** 10.1038/s41416-019-0633-0

**Published:** 2019-11-19

**Authors:** Guanghui Zhang, Qingzong Zhu, Gang Fu, Jianbing Hou, Xiaosong Hu, Jiangjun Cao, Wen Peng, Xiaowen Wang, Fei Chen, Hongjuan Cui

**Affiliations:** 1 State Key Laboratory of Silkworm Genome Biology, Southwest University, Chongqing, 400715 China; 2 Chongqing Engineering and Technology Research Centre for Silk Biomaterials and Regenerative Medicine, Southwest University, Chongqing, 400715 China; 3 Engineering Research Centre for Cancer Biomedical and Translational Medicine, Southwest University, Chongqing, 400715 China; 4 Cancer Center, Medical Research Institute, Southwest University, Chongqing, 400715 China; 5 Dental Hospital Affiliated to Chongqing Medical University, Chongqing, 400016 China; 6 Department of Pharmaceutical Sciences EACPHS, Wayne State University 259 Mack Avenue, Detroit, MI 48201 USA

**Keywords:** Oncogenes, CNS cancer

## Abstract

**Background:**

Thyroid hormone receptor interactor 13 (TRIP13) is an AAA + ATPase that plays an important role in the mitotic checkpoint. TRIP13 is highly expressed in various human tumours and promotes tumorigenesis. However, the biological effect of TRIP13 in GBM cells remains unclear.

**Methods:**

We generated GBM cell models with overexpressed or silenced TRIP13 via lentivirus-mediated overexpression and RNAi methods. The biological role of TRIP13 in the proliferation, migration and invasion of GBM cells has been further explored.

**Results:**

Our research indicated that TRIP13 was highly expressed in GBM tissues and cells. We found that the proliferation, migration and invasion abilities were inhibited in TRIP13-knockdown GBM cells. These results indicated that TRIP13 plays an important role in the tumorigenesis of GBM. Moreover, we found that TRIP13 first stabilised c-MYC by inhibiting the transcription of FBXW7, which is an E3 ubiquitin ligase of c-MYC, by directly binding to the promoter region of FBXW7. Therefore, our study indicated that the TRIP13/FBXW7/c-MYC pathway might provide a prospective therapeutic target in the treatment of GBM.

**Conclusions:**

These results indicated that TRIP13 plays an oncogenic role in GBM. The TRIP13/FBXW7/c-MYC pathway might act as a prospective therapeutic target for GBM patients.

## Background

Glioblastoma (GBM) is the most fatal and aggressive primary brain tumour, accounting for ~50% of all brain tumours.^1^ Glioblastoma, grade IV astrocytoma, is characterised by rapid proliferation and increased vascular and thrombosis formation,^2^ and it is accompanied by an extraordinarily poor prognosis. At present, the average survival time of GBM patients is ~15 months, and the level of treatment for patients is limited to achieving remission.^3,4^ Although treatments for GBM have made great progress in genetics and biomedicine, the survival rate of patients is still relatively low.^5^ Therefore, further exploration of pathogenesis and searching for new therapeutic targets is urgently needed for the treatment of GBM.

Thyroid hormone receptor-interacting factor 13 (TRIP13) exhibits conserved expression in many species, since it is an ATPase family member and functions in a variety of cellular-active protein families.^6^ TRIP13 was first identified as a protein interacting with the human papillomavirus E1 protein.^7^ Recent reports show that TRIP13 plays a significant role in meiotic recombination and DNA repair in plants, worms and mice,^8–12^ and it was also reported to be a newly discovered component of the spindle assembly checkpoint (SAC) pathway,^13–16^ which plays an important role in the distribution of chromosomes.^17^ High expression of TRIP13 has been found in various human cancers, including ovarian cancer, prostate cancer, colorectal cancer, hepatocellular carcinoma, lung adenocarcinoma and head and neck cancer. Abnormal expression of TRIP13 might be related to the development and occurrence of tumours, and the overexpression of TRIP13 can promote the connection of non-homologous terminals and increase the resistance to chemotherapeutic drugs.^18^ However, the molecular mechanism and biological consequences of TRIP13 in GBM cells are not clear.

C-MYC was initially identified as a cell homologue of a retroviral oncogene,^19,20^ and it is activated in 50% of human cancers;^21^ further, it can promote the proliferation of cancer cells by regulating 10–15% of the genes in the human genome.^22^ Reports show that c-MYC is overexpressed in many human cancers and can also regulate cell cycle and cell metabolic processes.^23^ Abnormal expression of c-MYC will lead to tumorigenesis. Therefore, the stability of the c-MYC protein is strictly controlled by FBXW7, which is an E3 ubiquitin ligase involved in ubiquitination and degradation of various carcinogenic substrates that contain F-box and WD40 repeat domains.^24^ Many studies have shown that abnormal expression of FBXW7 is the main cause of tumorigenesis.^25–27^ Inactivation of FBXW7 expression will enhance the proliferation and migration of tumours.^28^ Clinical data show that decreased expression of FBXW7 leads to poor prognosis in patients with gastric cancer,^29^ colorectal cancer^30^ and cholangiocarcinoma cancer.^31^ Deregulation of FBXW7 expression is reported to give rise to upregulation of c-MYC expression and to correlate with a poor prognosis in cancer patients.^29^

In this study, our experimental results showed that TRIP13 promoted cell proliferation, migration and invasion by regulating c-MYC stability in GBM cells. Mechanistically, we first found that the downstream target of TRIP13 is FBXW7, which is an E3 ubiquitin ligase involved in the ubiquitination of c-MYC. TRIP13 could inhibit FBXW7 expression to regulate c-MYC levels. Taken together, these data demonstrated that TRIP13 promoted GBM cell tumorigenesis, migration and invasion through the TRIP13/FBXW7/c-MYC axis, and they identify a promising therapeutic target in the treatment of GBM.

## Materials and methods

### Reagents and antibodies and clinical tissue samples

MG132 and CHX were obtained from Sigma (Shanghai, China). Anti-TRIP13, anti-c-MYC, anti-FBXW7, anti-MMP7 and anti-HA were purchased from Proteintech (Wuhan, China). Mouse monoclonal anti-GAPDH was obtained from Beyotime (Shanghai, China). Anti-P21, anti-CDK4 anti-CCND1, anti-β-catenin, anti-E-cadherin, anti-N-cadherin and anti-Flag were obtained from Cell Signaling Technology (Shanghai, China). Anti-Ki67 and propidium iodide (PI) were purchased from BD Biosciences. The clinical tissue samples were purchased from Chaoying Biotechnology Co., Ltd. (Xian, China) and they were originally obtained from Tongxu County People’s Hospital of Henan Province.

### Transfection and infection experiments and plasmids

Small-hairpin shRNAs for TRIP13 and FBXW7 and a negative control shRNA (shGFP) were obtained from Gene Pharma Co. Ltd. (Shanghai, China) and were inserted into the pLKO.1 vector. The ubiquitination plasmid that contained a HA tag was purchased from Addgene (Beijing, China). The recombinant plasmid containing the human TRIP13 full-length cDNA cloned into the PCDH-CMV-MCS-EF1-Hygro vector was purchased from Youbao Company (Changsha, China). For transfection and infection experiments, the target plasmid and packaging plasmid were transfected into 293FT cells by using the transfection reagent Lipofectamine 2000 (Invitrogen, Carlsbad, CA, USA). Lentiviruses were collected 48 h later and were used to infect GBM cells twice, 12 h per infection. The infected cells were screened by treatment for 36 h with puromycin and hygromycin B, and the surviving cells were frozen and stored in liquid nitrogen for subsequent experiments. All the primers of shRNA sequences are given in Table 1.

**Table 1:** Primers of shRNA

shTRIP13-forward (5′−3′)	CCGGGCTACTCAACAGACATAATATCTCGAGATATTATGTCTGTTGAGTAGCTTTTTG
shTRIP13-reverse (5′−3′)	AATTCAAAAAGCTACTCAACAGACATAATATCTCGAGATATTATGTCTGTTGAGTAGC
shFBXW7-forward (5′−3′)	CCGGCCAGAGAAATTGCTTGCTTTACTCGAGTAAAGCAAGCAATTTCTCTGGTTTTTG
shFBXW7-reverse (5′−3′)	AATTCAAAAACCAGAGAAATTGCTTGCTTTACTCGAGTAAAGCAAGCAATTTCTCTGG

### Immunohistochemistry staining

Paraffin-embedded tumours were cut into slices with a thickness of 5 mm, and then the paraffin sections were dewaxed and hydrated. Then, paraffin slices were put into citrate buffer (pH 6.0) and heated in a microwave oven to 95 °C for 20 min to facilitate antigen retrieval. Then, endogenous peroxidase activity was quenched, which was followed by blocking with normal goat serum. Then, the TRIP13, Ki67, c-MYC and FBXW7 antibodies were diluted with PBS (1:200), and the antibodies were added to the paraffin sections and incubated overnight at 4 °C. Then, a horseradish peroxidase-linked secondary antibody was added and incubated with the sections, which was followed by the addition of a DBA reagent. The results were observed under a microscope before counterstaining with haematoxylin.

### Quantitative and reverse transcriptional PCR

The total RNA of cells was extracted by using TRIzoL reagent. Then, 2 µg of RNA was reverse transcribed into cDNA. The normalised expression control was based on the glyceraldehyde-3-phosphate dehydrogenase value. Finally, the expression of the mRNA was determined as the CT value. All quantitative primers are given in Table 2.

**Table 2:** RT-PCR primers

TRIP13-forward (5′−3′)	ACTGTTGCACTTCACATTTTCCA
TRIP13-reverse (5′−3′)	TCGAGGAGATGGGATTTGACT
c-MYC-forward (5′−3′)	AATAGAGCTGCTTCGCCTAGA
c-MYC-reverse (5′−3′)	GAGGTGGTTCATACTGAGCAAG
P21-forward (5′−3′)	CCAACAAACTTAACGTGCCAC
P21-reverse (5′−3′)	AGGCTCAACAGTAACTGCATC
CDK4-forward (5′−3′)	AAACTTGGAAATCCCGAGATTGC
CDK4-reverse (5′−3’)	CGAAACCAGTTCGGTCTTTCAA
CCND1-forward (5′−3′)	CAATGACCCCGCACGATTTC
CCND1-reverse (5′−3′)	CATGGAGGGCGGATTGGAA
FBXW7-forward (5′−3′)	ACTGGGCTTGTACCATGTTCA
FBXW7-reverse (5′−3′)	TGAGGTCCCCAAAAGTTGTTG

### Cell proliferation detection

To examine the proliferation ability of cells, 1×10^3^ cells were cultured in 96-well plates for 6 days. An MTT assay was used to detect cell viability and growth curves. All experiments were independently performed three times.

### BrdU staining

In total, 2 × 10^4^ cells were cultured in 24-well plates for BrdU staining experiments. The cells were incubated for 35 min with 10 µg/ml BrdU. The cells were fixed with 4% paraformaldehyde (PFA) for 20 min. Cells were treated with 1 mol/L HCL and blocked with 5% goat serum and 0.3% BSA for 2 h. Then, the cells were incubated with a primary antibody against BrdU (Abcam, Cambridge, MA, USA) overnight at 4 °C. Then, an Alexa Fluor^®^ 594 secondary antibody (H + L; Invitrogen) was incubated with the cells at room temperature for 2 h, and nuclear staining was then performed by incubating with DAPI (300 nM). Finally, the BrdU incorporation rate was calculated from at least ten randomly chosen microscopic fields.

### Flow cytometry

For the analysis of the cell cycle, the cells were collected and fixed with 70% ethanol overnight at 4 °C. The cells were incubated with potassium iodide (PI) for 30 min. Next, the cells were analysed by flow cytometry (BD Biosciences, San Jose, CA, USA). Experimental results were analysed with Cell Quest software (BD Biosciences).

### Migration, invasion and wound-healing assay

Experiments on cell migration and invasion were performed by using Transwell Chambers (8-µm pore size, Corning, Beijing, China). For the invasion experiment, the membranes were covered with Matrigel (BD Biosciences). DMEM with 10% foetal bovine serum was added under the chamber, and cells in serum-free DMEM were added to the upper chamber. After 48 h of culture, the cells were fixed with 4% paraformaldehyde for 20 min and then stained with crystal violet. The mean numbers of cells were calculated from at least six randomly chosen microscopic images. For the cell wound-healing assay, cells were cultured in a six-well plate, and wounds were made with a 10-µl pipette tip. Finally, the healing process of cells was observed by microscopy.

### Western blot assay

Cells were lysed with RIPA lysis buffer to obtain protein. The protein was separated on 12% gels by sodium sulfate polyacrylamide gel electrophoresis and was then transferred to a polyvinylidene difluoride membrane. The membrane was blocked with 5% BSA at room temperature for 2 h. The polyvinylidene fluoride membrane was incubated with a diluted primary antibody overnight at 4 °C. Then, the polyvinylidene fluoride membrane was incubated with the secondary antibody (peroxidase-labelled anti-mouse and anti-rabbit antibodies) at room temperature for 2 h. Finally, the results were analysed with the ECL Prime Western blotting (WB) detection system (GE Healthcare).

### Soft agar assay

A 0.6% agarose medium with a low melting point was added to the bottom of a six-well plate, and then 0.3% agarose medium with 1000 cells was laid on the top. After 15–20 days, the results were photographed and recorded by optical microscopy.

### Ubiquitination and turnover assay

For ubiquitination assays, shGFP, TRIP13 and HA plasmids were transfected into 293FT cells by using the transfection reagent Lipofectamine 2000 (Invitrogen, Carlsbad, CA, USA). After 48 h of transfection, 50 µg/ml proteasome inhibitor MG132 was added to the cells and incubated for 7 h. Cells were collected and lysed with RIPA lysis buffer for western blot and IP assays. For the turnover assay, the infected cells were screened for 36 h with puromycin and hygromycin B, and the surviving cells were treated with CHX at a concentration of 50 µg/ml. Then, cells were collected, lysed and analysed by western blot.

### Animal experimental procedures and tumour xenograft experiment

Animal experiments were approved by the Committee for Animal Protection and Utilization of Southwest University. All experiments were conducted in accordance with the Guidelines for Animal Health and Use (Ministry of Science and Technology, China, 2006). Four-week-old female nude mice were purchased from Huafukang Biotechnology Co., Ltd. (Beijing, China) and were placed in SPF rooms for feeding and observation. The mice were randomly divided into three groups. GBM cell lines (LN229) (1 × 10^6^ cells) stably transfected with shGFP, shTRIP13 and shTRIP13/TRIP13 were injected into subcutaneous tissue of the mouse on 18 February, 2019. To reduce the pain to the mice, a system was used to introduce nasal anaesthesia (isoflurane) before the subcutaneous injections. The advantages of isoflurane anaesthesia for animals are that they enter an anaesthetised state faster and recover quickly. Once the anaesthesia was stopped, the animals generally recovered within 2 min. The depth of anaesthesia was easy to control. If an animal was found to be in poor condition during the operation, the anaesthesia was immediately stopped, or mice were quickly oxygenated to rescue them. Therefore, animal safety was very good. Isoflurane did not affect metabolism in the body and was almost completely discharged from the alveoli by breathing, which had no effect on the experimental results; further, these conclusions regarding isoflurane are widely recognised internationally. The concentration of isoflurane was MAC 1.6%. The mouse anaesthesia system was purchased from Reyward Life Technology Co., Ltd. (Shenzhen, China). All experiments were performed on a sterile workbench of an SPF room on the first floor of the National Key Laboratory of Silkworm Genome Biology at Southwest University. The mice were sterilised with 75% medical alcohol after subcutaneous injection. The mice were observed every 3 days and weighed. The feeding conditions were strictly standardised. Before the tumours were collected, the previously described system was used to introduce nasal anaesthesia (isoflurane) into mice to reduce their pain. Then, the mice were killed by cervical dislocation, and the tumours were removed. The bodies of mice were frozen at - 20 °C before transferring the bodies to Laibite Biotech Inc. (Chongqing, China) for incineration. The weight of the mice and the volume of the tumours were measured every 3 days after the growth of the tumours began. The formula for calculating the volume of tumours was as follows: V = (length × width^2^)/2. Finally, the tumours were collected and photographed for subsequent immunohistochemical experiments.

### Luciferase reporter assay

The promoter region of FBXW7 was ligated into a pGL3 vector via polymerase chain reaction. The empty pGL3-basic vector and pRL-TK internal control vector were instantaneously transfected into 293FT cells as a negative control. Then, the pGL3 plasmid, the pRL-TK internal control vector (Promega) and a shTRIP13/TRIP13 vector were co-transfected into 293FT cells by transfection with Lipofectamine 2000. After 48 h of cell culture, luciferase reporter assays were performed according to the manufacturer’s instructions (Promega). There were three replicate experiments in each group.

### Chromatin immunoprecipitation

A chromatin immunoprecipitation (ChiP) assay was performed by using a ChiP assay kit (Millipore) according to the manufacturer’s instructions. Briefly, the Flag-TRIP13 vector was transfected into 293FT cells with Lipofectamine 2000. After 48 h, 293FT cells were cross-linked and lysed, and DNA was sheared into 200–800-bp fragments by using sonication. Precleared chromatin was immunoprecipitated with a Flag antibody obtained from Cell Signaling Technology (Shanghai, China), and after reversing the cross-linking, DNA was isolated for quantitative real-time PCR (qRT-PCR). The relevant primer sequences are presented in Table 3.

**Table 3:** ChiP experimental primers

FBXW7-1/-436-F	GTGCATAGATTGCCTTCCCAG
FBXW7-1/-436-R	CCATTCACAGTGCTCAATCAACTAT
FBXW7-372/-607-F	GACTGGCTGTTGGAAGAAGAAAATA
FBXW7-372/-607-R	ACGGCCTAAGATAAAGTCTGGAGAT
FBXW7-562/-835-F	GCCACTTTGAAGAGAGTCTTCATCT
FBXW7-562/-835-R	AAGCATAACAGTCACCCAACTGATT
FBXW7-804/-1029-F	TGTCTTTAATCAGTTGGGTGACTGT
FBXW7-804/-1029-R	ATGAGCACTATTTTCAAGTGTGTGC
FBXW7-1001/-1399-F	GAGAGCACACACTTGAAAATAGTGC
FBXW7-1001/-1399-R	AGTAATGTGAACACAACCAAAGCAG
FBXW7-1294/-1620-F	AAGGGACCTTACAGCACAGCC
FBXW7-1294/-1620-R	CTCCTCTTGGTTGACGAATACTCTC

### Patient data analysis

Patient data and gene expression datasets were obtained from R2: microarray analysis and visualisation platform (http://hgserver1.amc.nl/cgi-bin/r2/main.cgi). Kaplan-Meier analysis was performed, and the resulting survival curves were generated by using GraphPad Prism (version 6.0). All cut-off values for separating high and low expression groups were determined by the online R2 database algorithm.

### Statistical analysis

All observations were confirmed by at least three independent experiments. Quantitative data are expressed as the mean ± standard deviation. Two-tailed Student’s *t* tests were performed for paired samples. *P* < 0.05 was considered statistically significant.

## Results

### High expression of TRIP13 is associated with poor GBM patient prognosis

**Fig. 1:**
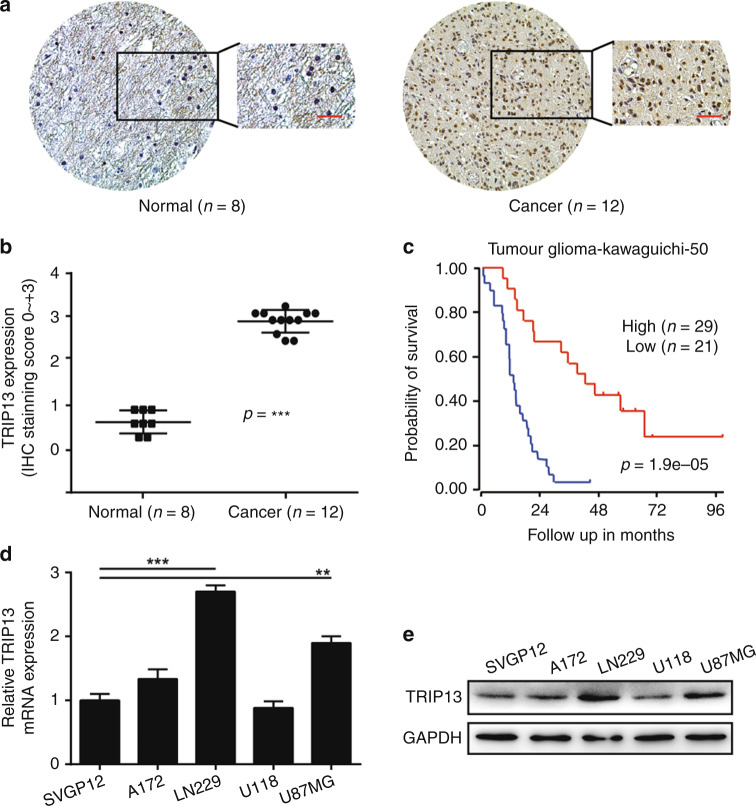
High expression of TRIP13 is associated with poor GBM patient prognosis. **a** Immunohistochemical staining analysis showed the expression of TRIP13 in 8 paired normal brain tissues and 12 paired GBM tissue samples. **b** Immunohistochemistry analyses is shown for TRIP13 expression levels in 8 paired normal brain tissue samples and 12 paired GBM tissue samples, *P* < 0.001. **c** Kaplan–Meier analysis of progression-free survival data from the Tumour Glioma Kawaguichi-50 database with the log-rank test *P*-values indicated. **d**, **e** Quantitative PCR assays and western blot assays were performed to detect the expression of TRIP13 in normal astrocytes (SVGP12) and GBM cell lines (A172, LN229, U118 and U87MG)

To verify that poor prognosis correlates with high expression of TRIP13 in GBM patients, an immunohistochemistry assay was performed to detect TRIP13 expression in normal tissues and GBM patient samples. The results indicated that the expression of TRIP13 was higher in GBM patient samples than in normal tissues (Fig. 1a, b). Then, the R2 gene database was used to analyse the relationship between TRIP13 expression and patient prognosis, and the results showed that high expression of TRIP13 was associated with poor prognosis in GBM patients (Fig. 1c). Subsequently, TRIP13 mRNA and protein expression were examined in normal astrocytes (SVGP12) and GBM cell lines by quantitative PCR and western blot experiments. The results demonstrated that TRIP13 was highly expressed in the U87MG and LN229 cell lines (Fig. 1d, e). Taken together, these data suggest that TRIP13 is significantly overexpressed in GBM and that TRIP13 might play an oncogenic role related to the poor prognosis of GBM patients.

### TRIP13 is required for the proliferation of GBM cells

**Fig. 2:**
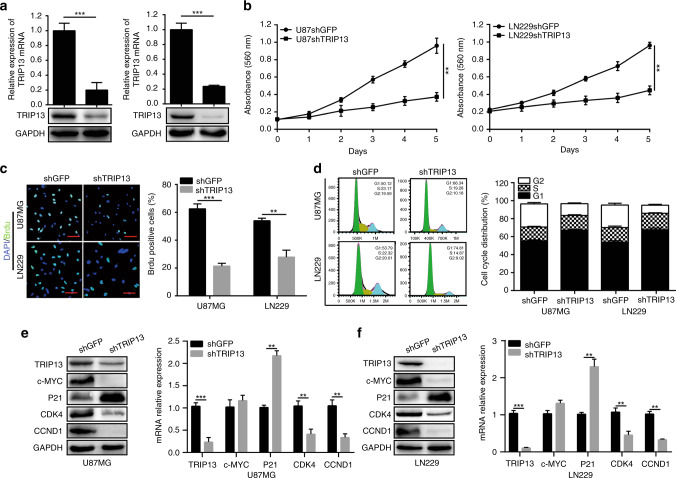
TRIP13 is required for the proliferation of GBM cells. **a** Western blot and quantitative PCR assays were used to characterise the expression of TRIP13 in TRIP13-knockdown U87MG and LN229 cells. **b** MTT assay was performed to examine the proliferative ability of TRIP13-knockdown U87MG and LN229 cells. **c** BrdU incorporation assays were performed to detect the amount of DNA synthesis in TRIP13-knockdown U87MG and LN229 cells. **d** The cell cycle was analysed in U87MG and LN229 cells by flow cytometry. **e**, **f** Western blot and quantitative real PCR assays were used to detect the expression of cell cycle-related proteins and mRNA levels in TRIP13-knockdown U87MG and LN229 cells

To further explore the effect of TRIP13 on the proliferation of GBM cells, we successfully knocked down TRIP13 expression in U87MG and LN229 cells by treating them with lentiviruses carrying shRNA sequences (Fig. 2a). Next, we examined the proliferation abilities of U87MG and LN229 cells with an MTT assay, and the results revealed that knocking down TRIP13 significantly inhibited the growth of GBM cells (Fig. 2b). BrdU incorporation experiments also showed that the amount of DNA synthesis in TRIP13-knockdown cell lines was obviously reduced compared with what was observed in control groups (Fig. 2c). Then, flow cytometry was used to detect the effect of TRIP13 on the cell cycle. We found that TRIP13 knockdown induced cell cycle arrest at the G1 phase (Fig. 2d). To further verify these results, G1 phase-related proteins were analysed by western blot and quantitative PCR assays. The results demonstrated that the protein and mRNA expression of CDK4 and CCND1 was reduced in TRIP13-knockdown GBM cells and that the expression of P21 was increased. However, the mRNA expression of c-MYC was not significantly changed in TRIP13-knockdown GBM cells (Fig. 2e, f). To further verify that the influence of TRIP13 knockdown on GBM cells is not caused by a mistarget effect, western blot assays were performed. The results showed that restoration of TRIP13 expression could partially rescue the expression of G1-related proteins. Then, we performed MTT and BrdU incorporation assays by using GBM cells, and the results showed that overexpression of TRIP13 could rescue the proliferation abilities of TRIP13-knockdown GBM cells (Supplementary Fig. 1a, b, c). Furthermore, flow cytometry analysis showed that overexpression of TRIP13 could obviously rescue the cell cycle defect (Supplementary Fig. 1d). Taken together, these results showed that TRIP13 promoted the proliferation of GBM cells.

### TRIP13 promotes the migration and invasion of GBM cells

**Fig. 3:**
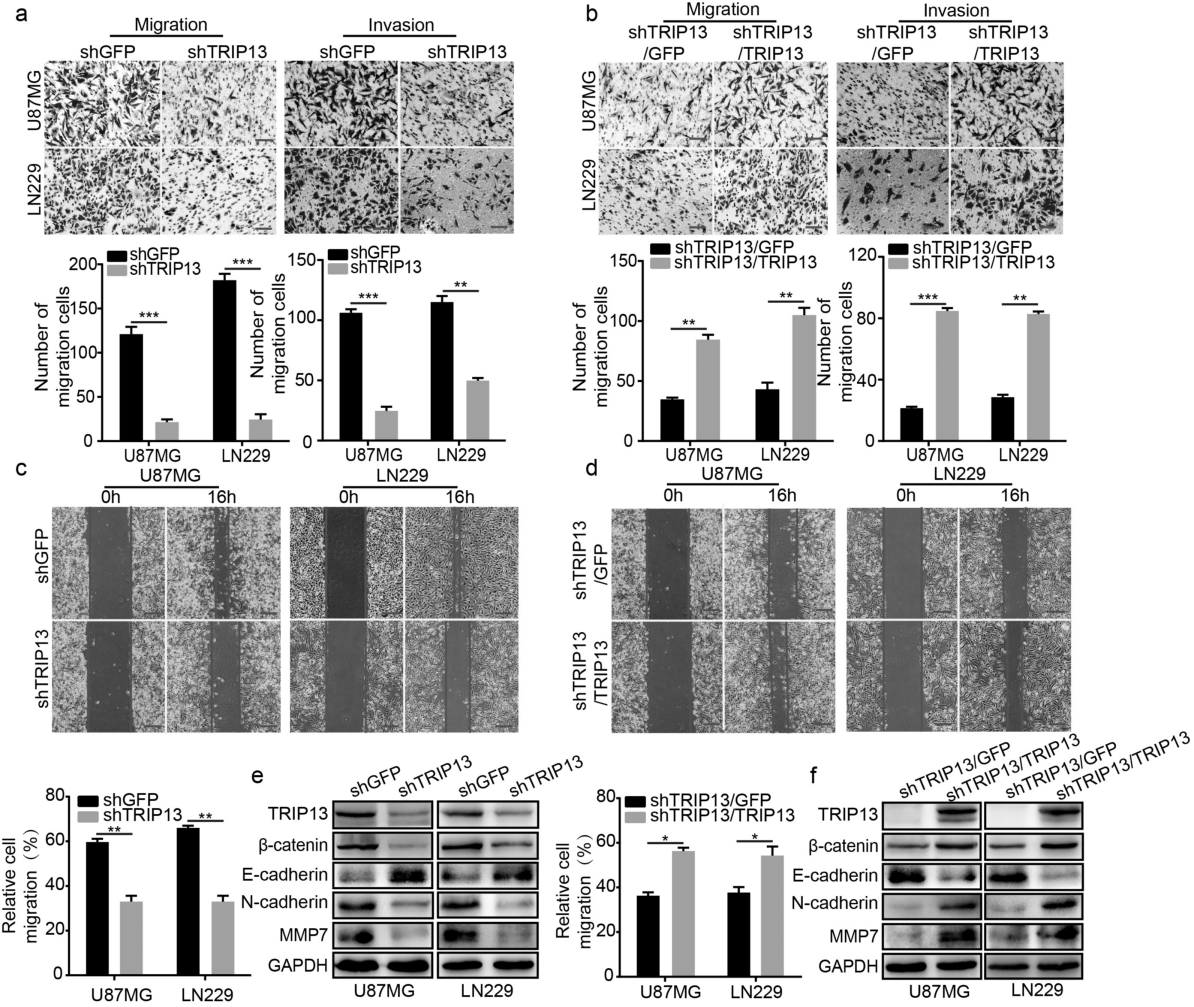
TRIP13 promotes the migration and invasion of GBM cells. **a** Migration and invasion assays were performed with TRIP13-knockdown U87MG and LN229 cells. **b** Wound-healing assay was performed with TRIP13-knockdown U87MG and LN229 cells. **c** Western blot analysis was performed to detect the expression of metastasis-related proteins in TRIP13-knockdown cells. **d**, **e** Migration, invasion and wound-healing experiments were performed after TRIP13 was rescued in TRIP13-knockdown U87MG and LN229 cells and negative controls. **f** Western blot analysis was performed to detect the expression of metastasis-related proteins after overexpression of TRIP13 in TRIP13-knockdown cells. All data are shown as the mean ± SD, *P < 0.05, **P < 0.01, ***P < 0.001

Glioblastoma (GBM) is a highly malignant and invasive primary brain tumour. To verify whether TRIP13 promoted the migration and invasion of GBM cells, a Transwell assay was performed with U87MG and LN229 cells. The results demonstrated that GBM cells with TRIP13 knocked down migrated and invaded much more slowly than the control cells (Fig. 3a). Subsequently, the wound-healing assay indicated that the migratory ability of GBM cells with TRIP13 knocked down was significantly lower than that of the control cells (Fig. 3c). Western blot assays were further used to verify the role of TRIP13 in the migration and invasion of GBM cells, and the protein expression levels of β-catenin, N-cadherin and MMP7 were significantly reduced, while the expression of E-cadherin was increased, and these proteins are markers of migration and invasion, respectively (Fig. 3e). To further evaluate whether overexpression of TRIP13 could restore the migration and invasion abilities of GBM cells with TRIP13 knocked down, Transwell assay, wound-healing assay and western blot assay were performed, and the results indicated that the migration and invasion ability of GBM cells was significantly rescued (Fig. 3b, d, f). Taken together, these experimental data indicated that TRIP13 plays an indispensable role in the migration and invasion of GBM cells.

### TRIP13 is required for colony formation and tumorigenesis of GBM cells

**Fig. 4:**
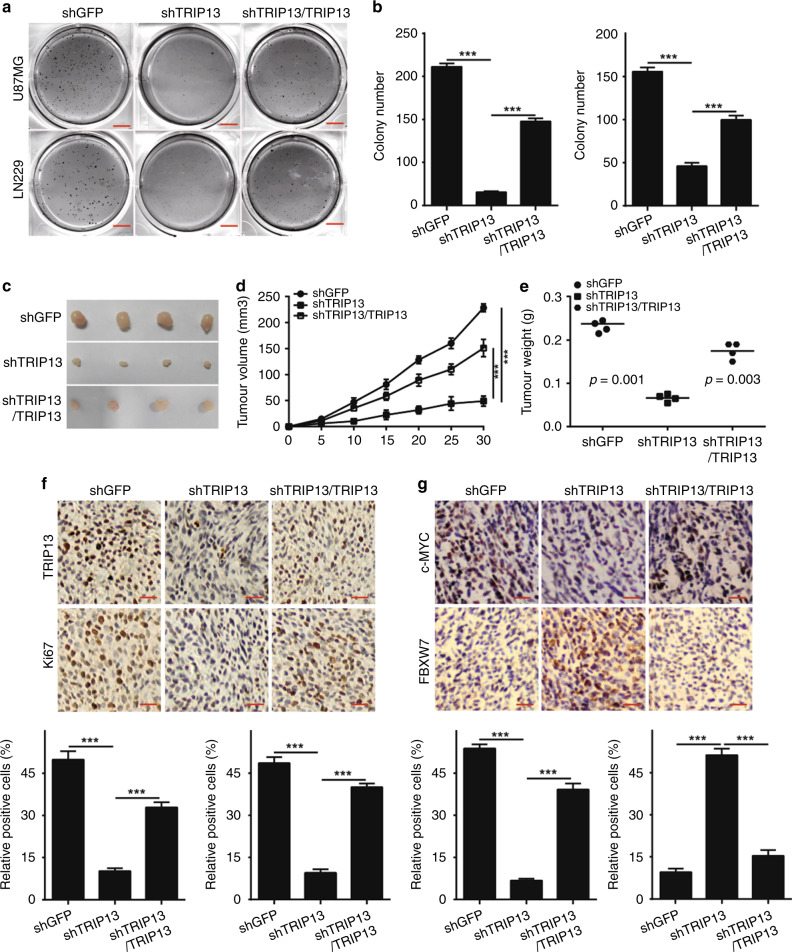
TRIP13 is required for colony formation and tumorigenesis of GBM cells. **a**, **b** Soft agar assay was performed in U87MG and LN229 cells with TRIP13 knocked down as well as TRIP13-rescued TRIP13-knockdown cells. Quantification of the number of cell clones. **c**, **e** The size and weight of xenograft tumours were analysed in TRIP13-knockdown and TRIP13-rescued TRIP13-knockdown LN229 cells. **d** The growth curve of tumours was determined for TRIP13-knockdown and rescue of TRIP13-knockdown LN229 cells that were injected into immunodeficient mice. These data were analysed with a two-tailed Student’s *t* test, and the *P*-value is indicated. **f**, **g** Immunohistochemical staining was performed to detect the expression of TRIP13, Ki67, c-MYC and FBXW7 in TRIP13-knockdown and rescue of TRIP13-knockdown tumour tissues. All *P*-values are based on the control versus treatment group

To investigate the effect of TRIP13 on colony formation and tumorigenesis of GBM cells in vitro and in vivo, soft agar assays were performed, and they showed that the number and size of clones in TRIP13-knockdown GBM cells were significantly smaller than those of the controls (Fig. 4a, b). Xenograft experiments indicated that the growth rate of tumours and the volume and weight of tumours in TRIP13-knockdown GBM cells were significantly decreased compared with those in the control groups (Fig. 4c, d, e). Furthermore, immunohistochemical staining suggested that the expression of TRIP13, Ki67 and c-MYC was significantly reduced in TRIP13-knockdown tumours compared with controls, while the expression of FBXW7 was increased (Fig. 4f, g). Then, after overexpression of TRIP13 in TRIP13-knockdown GBM cells, the abilities of colony formation and tumorigenesis were partially rescued, and the expression of TRIP13, Ki67, c-MYC and FBXW7 was also restored (Fig. 4a–g). These data suggested that TRIP13 was indispensable for the cloning and tumorigenesis of GBM cells.

### TRIP13 regulates the stability of c-MYC by reducing c-MYC ubiquitination

**Fig. 5:**
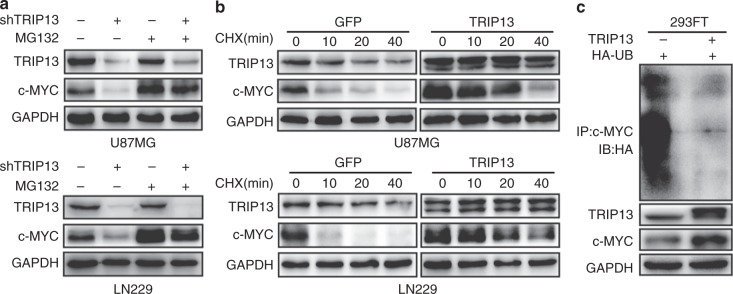
TRIP13 regulates the expression of c-MYC by reducing c-MYC ubiquitination. **a** Cell lysates were prepared from TRIP13-knockdown cells that had been treated with or without MG132 for 7 h. Equal amounts of cell lysates were immunoblotted with the indicated antibodies. **b** The c-MYC turnover rate of TRIP13-overexpressing cells is shown. U87MG and LN229 cells were transfected with TRIP13 plasmid and then treated with CHX (100 μg/ml) for the indicated times. Cell lysates were immunoblotted with the indicated antibodies. **c** Transfected 293FT cells were treated with MG132 for 7 h before proteins were harvested. The ubiquitinated c-MYC proteins were pulled down with an anti-c-MYC antibody and immunoblotted with an anti-HA antibody

Overexpression of c-MYC promotes GBM tumorigenesis. Previous studies have shown that the expression of c-MYC protein was downregulated in TRIP13-knockdown GBM cells. However, the mRNA levels of c-MYC were not significantly changed in TRIP13-knockdown cells (Fig. 2e, f). We speculated that c-MYC might be degraded by ubiquitination. To further confirm that TRIP13 regulates the ubiquitination of c-MYC, TRIP13-knockdown GBM cells were treated with MG132, and the results indicated that the protein expression of c-MYC was obviously rescued (Fig. 5a). Moreover, the de novo protein synthesis inhibitor cycloheximide (CHX) was used to examine the turnover rate of c-MYC, and we found that the degradation of c-MYC was decreased in TRIP13-overexpression groups (Fig. 5b). To further examine the ubiquitination effect of TRIP13 on c-MYC, a ubiquitination assay was performed in vitro, and it indicated that overexpression of TRIP13 could significantly decrease the ubiquitination level of c-MYC (Fig. 5c). In general, these results suggested that TRIP13 regulated the stability of c-MYC by decreasing the ubiquitination levels of c-MYC.

### TRIP13 regulates the ubiquitination of c-MYC through transcriptional inhibition of FBXW7

**Fig. 6:**
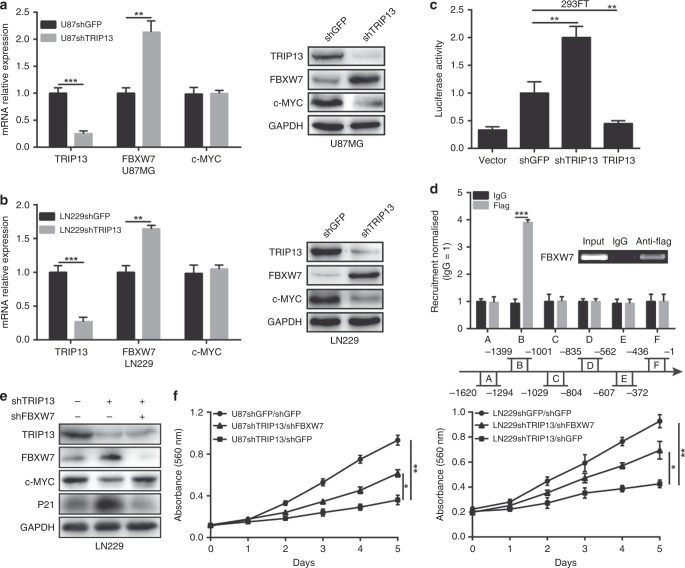
TRIP13 regulates the ubiquitination of c-MYC through FBXW7. **a**, **b** Western blot and quantitative PCR assays were performed to examine the protein and mRNA expression of c-MYC and FBXW7 in TRIP13-knockdown U87MG and LN229 cells. **c** The FBXW7 promoter regions were ligated into the pGL3 plasmid and co-transfected with pRL-TK/shTRIP13/TRIP13. Luciferase activity was examined 48 h after transfection. The pGL3-basic vector was used as the negative control. **d** A total of six sets of primers were designed within the human FBXW7 promoter, and a ChiP assay was performed by using Flag antibodies. IgG was used as the negative control. **e**, **f** Western blot and MTT experiments were performed to assess FBXW7 knockdown in LN229 and U87MG cells with knocked-down TRIP13. All data are shown as the mean ± SD, *P < 0.05, **P < 0.01, ***P < 0.001

FBXW7 is a well-known E3 ubiquitin ligase of c-MYC. However, TRIP13 is not an E3 ubiquitin ligase. We speculated that TRIP13 might reduce the level of c-MYC ubiquitination by regulating FBXW7. To further confirm our hypothesis, quantitative PCR and western blot assays were used to show that the expression of FBXW7 was significantly increased in TRIP13-knockdown GBM cells (Fig. 6a, b). Then, a dual-luciferase reporter assay was performed to determine the effect of TRIP13 on the FBXW7 promoter region. The results indicated that the promoter activity of FBXW7 was obviously enhanced in TRIP13-knockdown cells, and it was weakened in TRIP13-overexpressing cells (Fig. 6c). To further explore the transcriptional regulation of FBXW7 by TRIP13, a ChiP experiment was performed and showed that TRIP13-binding sites were enriched in the region (−1399 to −1001 bp) of the FBXW7 promoter (Fig. 6d). These results suggested that TRIP13 could inhibit FBXW7 transcription by directly binding to the promoter region of FBXW7. To further confirm that TRIP13 regulates c-MYC ubiquitination through FBXW7, western blot and MTT assays were performed to detect the protein expression and proliferation of TRIP13-knockdown GBM cells after FBXW7-knockdown treatment. The results indicated that the protein expression of c-MYC and P21 was partially restored, and the proliferation ability of TRIP13-knockdown cells was rescued after FBXW7-knockdown treatment (Fig. 6e, f). These results indicated that the TRIP13/FBXW7/c-MYC pathway might play an important role in the tumorigenesis of GBM.

## Discussion

Glioblastoma (GBM) is a highly malignant and invasive cancer that has an extremely poor prognosis. At present, the average survival time of GBM patients is short, and the optimal treatment for GBM patients has a limited ability to achieve remission.^3,4^ Therefore, it is urgent to seek molecular and drug targets for the treatment of GBM. TRIP13 is a thyroid hormone receptor-interacting factor, and it has been reported that abnormal expression of TRIP13 might be related to tumorigenesis and might lead to resistance to chemotherapeutic drugs.^18^ In addition, high expression of TRIP13 has been found in various human cancers. Many reports have indicated that TRIP13 plays a significant role in meiotic recombination and promotes the tumorigenesis of human cancers.^8–12^ However, the biological functions of TRIP13 in GBM cells remain unclear.

Our data showed that TRIP13 was highly expressed in GBM tissues and cells, and that the overexpression of TRIP13 was significantly correlated with a poor prognosis for GBM patients. Subsequently, by knocking down and rescuing the expression of TRIP13 in GBM cells, we found that TRIP13 can promote the proliferation, migration and invasion of GBM cells. Subcutaneous tumorigenesis experiments in mice showed that TRIP13 significantly promoted the growth of tumours in vivo. Furthermore, through cell cycle analysis, we found that the cell cycle was blocked in the G1 phase after knocking down TRIP13. Taken together, these results suggest that TRIP13 plays an indispensable role in the tumorigenesis of GBM.

Although TRIP13 is highly expressed in many human cancers, the biological molecular mechanism is largely unclear. Current reported studies show that TRIP13 can regulate several cancer-related factors, including TGF-β1, SMAD3, NOTCH and DNA-PKcs complex proteins.^18,32,33^ Our study showed that TRIP13 knockdown decreased the protein expression of c-MYC and its downstream molecules, CDK4 and CCND1, and the expression of P21 was increased in TRIP13-knockdown GBM cells. However, further studies showed that the total mRNA level of c-MYC was not significantly changed in TRIP13-knockdown GBM cells. Therefore, we suspected that TRIP13 might regulate the expression of c-MYC through ubiquitination degradation. FBXW7 is a well-known E3 ubiquitin ligase of c-MYC. Then, we examined the expression of FBXW7 in TRIP13-knockdown GBM cells. The results indicated that the expression of FBXW7 was significantly increased in TRIP13-knockdown cells. Dual-luciferase reporter assay and ChiP assay results showed that TRIP13 can regulate the transcription of FBXW7 by binding to the promoter region of FBXW7.

In general, these results indicated that TRIP13 promoted the proliferation, migration and invasion of GBM cells. Our study indicated for the first time that TRIP13 promoted the proliferation, migration and invasion of GBM cells via the activated FBXW7/c-MYC pathway. These results indicated that TRIP13 might provide a prospective therapeutic target in the treatment of GBM patients.

## Supplementary information

Supplementary data

## Data Availability

All the data analysed or generated in this study are included in this article and its Supplementary Information file.
