# Supplementary material for: TRIP13 promotes the cell proliferation, migration and invasion of glioblastoma through the FBXW7/c-MYC axis
Source: Br J Cancer. 2019 Nov 19;121(12):1069–78. doi: 10.1038/s41416-019-0633-0 (PMC6964669; doi:10.1038/s41416-019-0633-0)
Supplement: Supplementary file 1 — Supplementary data [file 41416_2019_633_MOESM1_ESM.docx]

Supplementary data


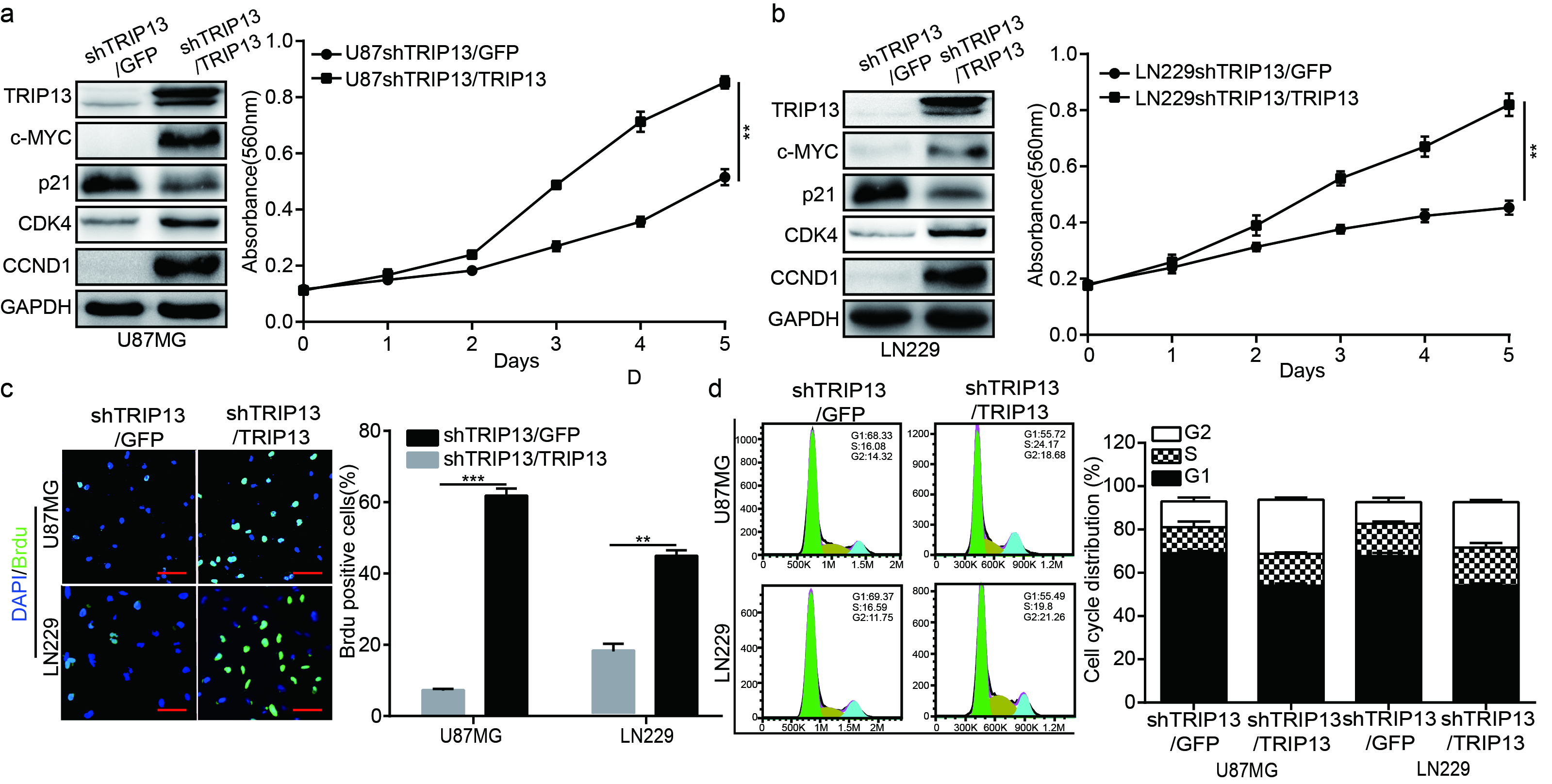


**Supplementary Fig. 1** Restoration of TRIP13 expression can partially rescue the proliferation abilities of GBM cells. **a, b** Western blot and MTT assays were performed to examine the expression of cycle-related proteins and cells viability after TRIP13 rescued in TRIP13-knockdown U87MG and LN229 cells. **c** BrdU incorporation assay was performed to detect the amount of DNA synthesis after TRIP13 rescued in TRIP13-knockdown U87MG and LN229 cells. **d** The cell cycle was analyzed after overexpression of TRIP13 in TRIP13-knockdown cells. All data are shown as the mean ± SD, *P<0.05, **P<0.01 ***P<0.001.
